# Coexistence of Carcinoma and Tuberculosis in the Cecum: A Clinical Conundrum

**DOI:** 10.7759/cureus.58675

**Published:** 2024-04-21

**Authors:** Sonali Mallik, Ananda Datta, Raghavendrun Sivasankar, Archana Malik

**Affiliations:** 1 Pulmonary Medicine, Institute of Medical Sciences and Sum Hospital, Bhubaneswar, IND; 2 Pulmonary Medicine, All India Institute of Medical Sciences, Deoghar, IND

**Keywords:** abdominal tuberculosis, carcinoma cecum, intestinal tuberculosis, colon cancer, coexisting tuberculosis and carcinoma

## Abstract

The coexistence of carcinoma of the colon and tuberculosis (TB) represents a rare and intricate clinical scenario. It poses significant challenges in both diagnosis and management. Clinical prediction of this coexistence is challenging since the clinical features of these two conditions are often similar. Likewise, the radiology is not decisive because of the significant overlap in the image findings of carcinoma and TB. A conclusive diagnosis relies on histopathological evidence of both malignancy and TB. Here, we report a case of a 58-year-old female who presented with chronic abdominal pain. Computed tomography showed the presence of a mass in the cecum. Histopathology of tissue retrieved through colonoscopy was indicative of features of both TB and adenocarcinoma of the cecum. *Mycobacterium tuberculosis* was detected in the tissue by cartridge-based nucleic acid amplification test. The patient was initiated on antitubercular treatment. She underwent surgical resection of the mass and is planned for adjuvant chemotherapy.

## Introduction

The simultaneous encounter of tuberculosis (TB) and malignancy within the same organ poses a unique and formidable challenge for the clinician from both diagnostic and therapeutic points of view. Abdominal TB constitutes approximately 13% of cases of extrapulmonary TB, with gastrointestinal TB representing a substantial proportion ranging from 43% to 65% of all abdominal TB cases [1]. The most involved site is the ileocecal junction due to anatomical and physiological vulnerability. The occurrence of cecal carcinoma is relatively uncommon, constituting only 8% of all cases of colon cancer [2]. The concurrent presence of TB and carcinoma in the colon is an infrequently observed phenomenon. Further, the presence of both pathologies within the same part of the colon is even rarer [3]. Here, we report a case of a 58-year-old female diagnosed with both TB and adenocarcinoma of the cecum simultaneously by colonoscopic biopsy.

## Case presentation

A 58-year-old woman presented with complaints of abdominal pain for two months. The pain was dull aching, continuous, and moderate in intensity without any radiation. She also complained of anorexia and weight loss. There was no history of fever, dysuria, vomiting, diarrhea, or blood in the stool.

On physical examination, her vital signs were as follows: pulse rate of 88 beats per minute, respiratory rate of 16 times per minute, blood pressure of 138/90 mmHg, and oxygen saturation at room air of 98%. Pallor was present, but there was no peripheral lymphadenopathy. Her abdomen was soft but deep tenderness was present in the right iliac fossa.

**Figure 1:**
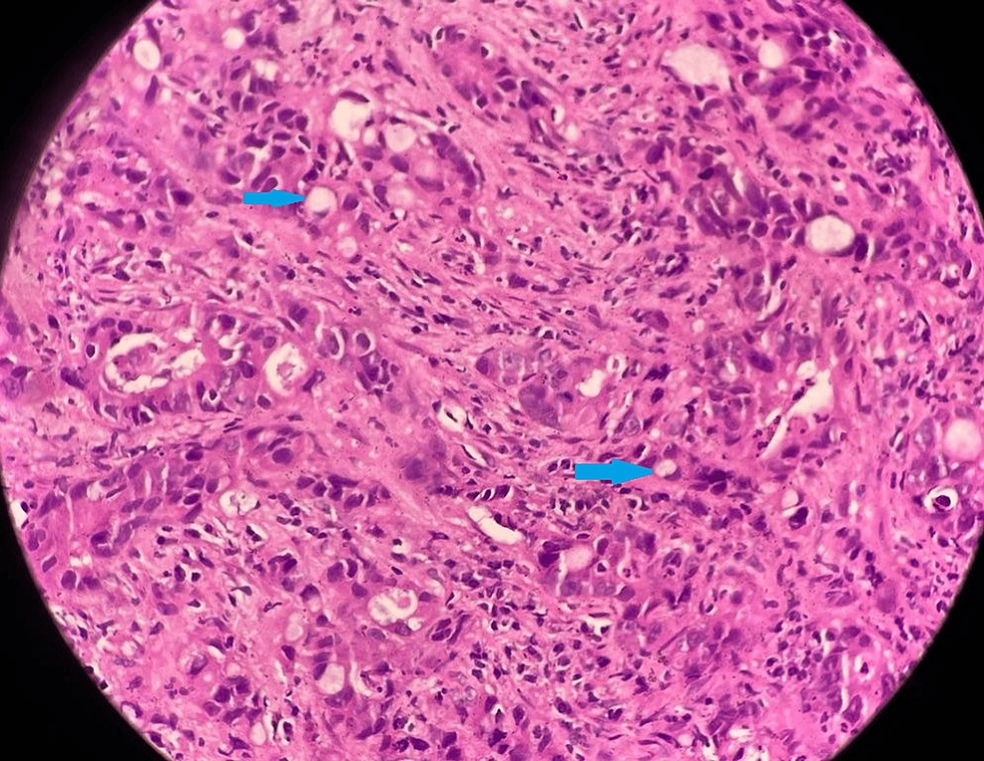
Histopathology of cecal mass biopsy (hematoxylin and eosin stained) showing pleomorphic tumor cells arranged in acinar configuration, cords, and trabeculae. There is moderate nuclear atypia and signet ring cell differentiation (blue arrows).

A complete hemogram revealed anemia with a hemoglobin level of 9.1 gm/dl. The total leucocyte count and platelet count were within normal ranges. The erythrocyte sedimentation rate was 101 mm in the first hour. Liver function tests were normal except for an alkaline phosphate level of 560 U/L. Viral markers were non-reactive. Chest X-ray was normal. Ultrasonography of the abdomen showed the presence of a cecal mass. Contrast-enhanced computed tomography (CECT) of the abdomen and pelvis revealed the presence of a heterogeneous mass in the cecum with circumferential wall thickening involving the terminal ileum and ileocecal junction. There was also the presence of multiple enlarged adjacent mesenteric lymph nodes and multiple tiny mesenteric deposits. CECT of the thorax showed no abnormality. The serum carcinoembryonic antigen level was 21 ng/ml (normal value: <5 ng/ml). A colonoscopy showed an irregular mass in the cecum, while the scope could not be negotiated further. A biopsy was taken from the cecal mass. Histopathology revealed an infiltrating neoplasm composed of pleomorphic tumor cells arranged in acinar configuration, cords, and trabeculae. There was moderate nuclear atypia and signet ring cell differentiation (Figure 1).

**Figure 2:**
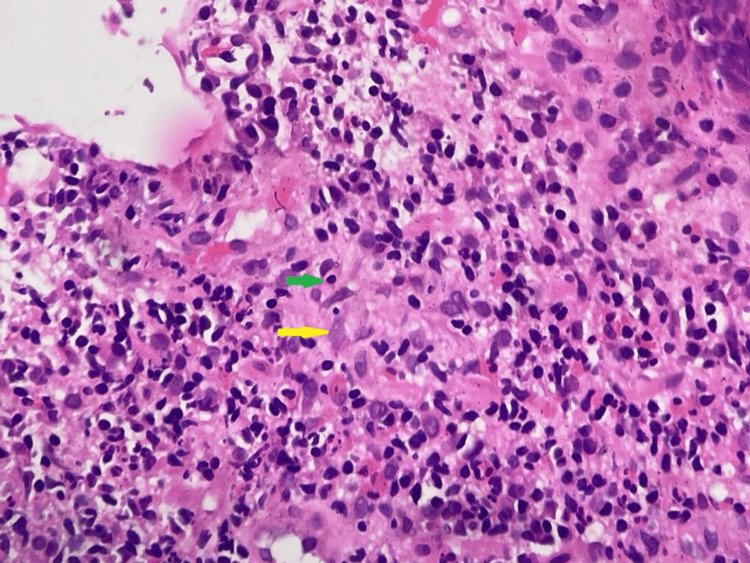
Histopathology of cecal mass biopsy (hematoxylin and eosin stained) showing infiltration of lymphocytes (green arrow) and epithelioid cells (yellow arrow) with the formation of a focus of poorly formed granuloma.

Immunohistochemistry showed that the tumor was mismatch repair protein proficient/microsatellite stable (pMMR/MSS). Also, the subjacent tissue showed ill-defined granuloma with features of chronic inflammation (Figure 2).

Cartridge-based nucleic acid amplification test of the tissue detected *Mycobacterium tuberculosis*, which was rifampicin sensitive. A final diagnosis of moderately differentiated adenocarcinoma of the cecum (stage IV) along with cecal TB was made. Anti-tuberculosis therapy (ATT) was started with a fixed-dose combination of rifampicin, isoniazid, ethambutol, and pyrazinamide according to the weight band as per national TB treatment guidelines. After one week, she underwent a right hemicolectomy. Intraoperatively, it was seen that a proliferative lesion measuring 6 x 8 cm was present in the cecum involving terminal ileum and proximal ascending colon. The mass was abutting the right ovary without any loss of planes. Multiple mesenteric lymph nodes of varying sizes were present along the right colic vessels and 12 lymph nodes were retrieved. The anterior surface of the liver was smooth, but the mesentery was studded with multiple tiny nodules. The proximal small bowel was not distended and the cecocolic lumen appeared to be patent. A primary ileocolic anastomosis was done. The intraoperative blood loss was around 170 ml and the operative duration was approximately three hours. She tolerated the procedure well and her postoperative period remained uneventful without any major complications. She was discharged after eight days. The pathological staging was T4N2M1c. Malignant involvement was seen in all the retrieved lymph nodes with three lymph nodes showing granuloma additionally. She is planned to start on adjuvant chemotherapy with a modified FOLFOX (5-fluorouracil, leucovorin, and oxaliplatin) regimen after six weeks of surgery.

## Discussion

Around 75 cases of simultaneous TB and carcinoma at the same site in the colon have been documented in the literature [3-16]. The details of the 18 available cases are summarized in Table 1.

**Table 1:** Cases with coexistence of carcinoma colon and colonic tuberculosis. mFOLFOX6: modified 5-fluorouracil, leucovorin, and oxaliplatin. ATT: anti-tuberculosis therapy.

Study	Number of cases	Site of dual pathology	Mode of diagnosis	Type of surgery	Cancer chemotherapy	Anti-tubercular therapy	Outcome
Barson et al. (1970) [4]	1	Transverse colon and sigmoid colon	Surgery	Transverse colectomy and resection of proximal sigmoid colon	Not defined	Started after surgery	Died in 3 weeks
Tandon et al. (1974) [5]	1	Cecum, ascending colon	Surgery	Extended right hemicolectomy	Not defined	Not defined	Not defined
Dorai et al. (1991) [6]	1	Sigmoid colon	Surgery	Anterior resection with partial cystectomy	Not defined	Started after surgery	Stable on 3 months follow up
Jain et al. (1991) [7]	2	a. Proximal transverse colon	Not defined	Segmental resection	Not defined	Not defined	Alive with carcinoma after 2.5 years follow-up
		b. Cecum	Not defined	Right hemicolectomy	Not defined	Not defined	Alive with carcinoma after 2.5 years follow-up
Sheikh et al. (1995) [8]	1	Cecum, ascending colon, and proximal transverse colon	Surgery	Right hemicolectomy	Not defined	Given; not specified	Alive with recurrence after 3 years
Isaacs et al. (1997) [9]	1	Cecum	Surgery	Right hemicolectomy	Not defined	Started 7 months before surgery and continued in the postoperative period	Died after 7 months
Singh et al. (2003) [10]	1	Ascending colon	Surgery	Left hemicolectomy	Not defined	Started after surgery; continued for 1 year	Recurrence-free after 3 years
Chakravartty et al. (2010) [11]	2	a. Cecum, terminal ileum, and ascending colon	Surgery	Limited colectomy	Not defined	Started before surgery	Not defined
		b. Cecum and ascending colon	Surgery	Right hemicolectomy	Not defined	Started after surgery	Not defined
Lin et al. (2014) [12]	1	Sigmoid colon	surgery	Anterior resection	mFOLFOX6 followed by capecitabine	Started after surgery	In stable condition after 2 years
Sharma et al. (2016) [13]	1	Cecum	Surgery	Right hemicolectomy	Not defined	Started before surgery	Not defined
Hossain et al. (2016) [14]	1	Descending colon	Surgery	Left hemicolectomy	Not defined	Not defined	Not defined
Ionescu et al. (2016) [15]	1	Sigmoid colon (fallopian tube also involved)	Surgery	Sigmoid colectomy and left adnexectomy with subtotal hysterectomy	Not defined	Started after surgery; continued for 1 year	Recurrence-free after 1 year
Chaudhary et al. (2021) [3]	3	a. Hepatic flexure	Not defined	Right hemicolectomy	Not defined	Not defined	Alive and recurrence-free at 2 years
		b. Transverse colon	Not defined	Extended right hemicolectomy	Not defined	Not defined	Alive and recurrence-free at 2 years
		c. Ascending colon	Not defined	Right hemicolectomy excision of infiltration to the lateral abdominal wall	Not defined	Not defined	Died at 9 months unrelated to primary disease
Park et al. (2022) [16]	1	Proximal ascending colon	Colonoscopy	Right hemicolectomy	Given; the regimen was not specified	Started 2 weeks before surgery	On cancer chemotherapy and ATT without any significant adverse effect

Most cases were diagnosed using surgical specimens. Cancer chemotherapy details are missing in all but one case. In one-third of the cases, anti-TB drugs were initiated only after surgery, while in four cases, they were started before surgery. No drug interactions or adverse effects related to anti-TB drugs were reported in any of the cases.

The pathogenic mechanism of the simultaneous occurrence of TB and carcinoma colon is a matter of debate. Coexistence might be a coincidence or might have a causal relationship. Malignancy can potentially initiate a new tubercular lesion or reactivate an existing one by disrupting the host immunity. Tumor antigens and peptides alter the environment of granuloma and allow TB bacilli to grow leading to reactivation [3]. The occurrence of mycobacterial infection is found to be three to five times higher in patients with malignancy compared to the general population [17]. Conversely, repeated and chronic cellular insults by tubercle bacilli may set genetic mutation leading to neoplastic transformation. It is well-established that pulmonary TB can induce carcinogenesis in the lungs [18]. Similarly, a potential association can be between ulcerative lesions in intestinal tuberculosis and the development of carcinomas at the same site.

It is quite difficult to predict coexistence clinically. Although computed tomography is a valuable tool for diagnosing both intestinal carcinoma and TB, there is significant overlap concerning radiological findings like bowel wall thickening, mesenteric lymphadenopathy, omental soft tissue, and ascites [16]. A conclusive diagnosis relies on the histopathological evidence of both malignancy and TB along with bacteriological evidence confirming tuberculosis. Diagnosis can be confirmed by obtaining a biopsy through either a colonoscopic or surgical approach.

There is insufficient data in the literature regarding the ideal duration of ATT administration before surgery for colon cancer. When pulmonary tuberculosis and lung cancer coexist, lung resection is deemed safe after a two-week course of ATT to ensure the individual reaches a non-infectious state. Since intestinal tuberculosis is not contagious, ATT can be prescribed either before or following colon surgery. Adjuvant cancer chemotherapy can be continued along with ATT [3,16].

Both anti-TB drugs and oxaliplatin-based regimens are notorious for causing gastrointestinal toxicity, hepatotoxicity, neuropathy, and hematological abnormalities. The combined use can potentially increase the chance as well as the severity of these adverse reactions. Oxaliplatin-based regimen is known to cause hepatic sinusoidal injury with an incidence ranging from 19% to 78%. The spectrum of manifestations ranges from cholestatic or hepatocellular acute liver failure to chronic non-cirrhotic portal hypertension [19]. Hepatotoxicity attributed to anti-TB drugs has been reported in 5%-28% of cases. There is a hepatocellular injury in the form of lobular hepatitis, sub-massive to massive necrosis, and hydropic degeneration of hepatocytes in severe cases [20]. Therefore, the concurrent administration of the FOLFOX regimen and ATT can amplify their hepatotoxic effects synergistically.

## Conclusions

The simultaneous occurrence of TB and malignancy in the colon presents a complex challenge for clinicians due to the rarity of incidence and overlapping clinicoradiological presentation. Further research is essential to elucidate the underlying mechanisms and establish standardized protocols for managing these dual pathologies to ensure optimal patient outcomes.
